# The RAFT Telemedicine Network: Lessons Learnt and Perspectives from a Decade of Educational and Clinical Services in Low- and Middle-Incomes Countries

**DOI:** 10.3389/fpubh.2014.00180

**Published:** 2014-10-07

**Authors:** Georges Bediang, Caroline Perrin, Rafael Ruiz de Castañeda, Yannick Kamga, Alexandre Sawadogo, Cheick Oumar Bagayoko, Antoine Geissbuhler

**Affiliations:** ^1^Department of Radiology and Medical Informatics, Faculty of Medicine, University of Geneva, Geneva, Switzerland

**Keywords:** telemedicine, eHealth, low and middle income countries, Africa, telemedicine network

## Abstract

**Background:** The objectives of this paper are to (i) provide an overview of the educational and clinical experiences of the Réseau en Afrique Francophone pour la Télémédecine (RAFT) network, (ii) analyze key challenges and lessons learnt throughout a decade of activity, and (iii) draw a vision and perspectives of its sustainability.

**Methods:** The study was carried out following three main stages: (i) a literature review, (ii) the analysis of key documents, and (iii) discussions with key collaborators of the RAFT.

**Results:** Réseau en Afrique Francophone pour la Télémédecine has been offering an important quantity of educational, clinical, and public health activities during the last decade. The educational activities include the weekly delivery of video-lectures for continuing and post-graduate medical education, the use of virtual patients for training in clinical decision making, research training activities using ICTs and other e-learning activities. The clinical and public health activities include tele-expertise to support health professionals in the management of difficult clinical cases, the implementation of clinical information systems in African hospitals, the deployment of mHealth projects, etc. Since 2010, the RAFT has been extended to the Altiplano in Bolivia and Nepal (in progress).

**Lessons Learnt and Perspectives:** Important lessons have been learnt from the accumulated experiences throughout these years. These lessons concern: social and organization, human resources, technologies and data security, policy and legislation, and economy and financing. Also, given the increase of the activities and the integration of eHealth and telemedicine in the health system of most of the countries, the RAFT network faces many other challenges and perspectives such as learning throughout life, recognition, and valorization of teaching or learning activities, the impact evaluation of interventions, and the scaling up and transferability out of Africa of RAFT activities. Based on the RAFT experience, effective integration and optimum use of eHealth and telemedicine in low- and middle-income countries (LMICs) health systems should take into account the context (resources, infrastructure, and funding), the needs of key stakeholders, and the results derived from theoretical and practical experience. The relevant items highlighted to illustrate the sustainability of the RAFT network and the analyses performed in this study, should serve as discussion basis for the development of eHealth and telemedicine in LMICs.

## Background

Health systems in Sub-Saharan low- and middle-income countries (LMICs) face particularly high burden of diseases with poor infrastructure and sanitary equipment, and scarce numbers of trained health professionals (1). These health professionals are mainly concentrated in urban areas and those in rural areas are professionally isolated (2). In addition, geographical and/or financial limitations make quality care widely inaccessible in these countries. Important distances must be covered in some cases to consult a specialist with not negligible risks and costs for the patient, especially considering the quality of the roads and transportation services in Africa.

Telemedicine and eHealth are increasingly used to improve quality and access to healthcare in LMICs (3–7). The Réseau en Afrique Francophone pour la Télémédecine (RAFT) network (8) was created in Africa more than a decade ago to support health professionals working in “medical deserts” (9–11).

This paper overviews the educational and clinical activities of the RAFT analyses lessons learnt throughout a decade of activity and draws perspective on future challenges and sustainability.

## RAFT Network

The RAFT (8) is a telemedicine network created by the University hospitals and the University of Geneva (HUG and UNIGE) in French-speaking Africa (Mali) in 2001. In 2008, it extended to English-speaking Africa and is currently being deployed in Portuguese-speaking Africa (Angola). The RAFT is primarily African with 13, 5, and 1, French, English, and Portuguese-speaking countries, respectively. However, it has become more global with the adhesion of Bolivia and Nepal since 2010 and 2014, respectively. The RAFT includes 60 active sites^1^ and it connects hundreds of health professionals around the world. Connections are generally established between central and university hospitals, and district hospitals. The management is decentralized with a local team in each country responsible for the strategy, the implementation, and the coordination of activities at the national level (10). A small central team in Geneva ensures the general coordination (10). The RAFT is a collaborating center of World Health Organization (WHO) for eHealth and telemedicine and collaborates with other institutions such as the Université Numérique Francophone Mondiale (UNFM).

## Methods

We used general and specific sources of information to understand the past, present, and future of the RAFT network and identify its difficulties, successes, and failures. First, we reviewed literature (scientific papers mostly) in search for equivalent initiatives to the RAFT in LMICs using PubMed and WHO Databases. Second, we reviewed reports, workshops proceedings, scientific papers and tools (software) to obtain specific quantitative and qualitative information on the RAFT activities (i.e., projects, participation, deployment status, etc.). Third, we held informal discussions with key experienced members (local and international coordinators, national focal points) of the RAFT.

## Activities

### Educational activities

#### Distant continuing medical education for healthcare professionals

The continuing medical education (CME) targets health professionals in remote areas and is based on live video-lectures of approximately 45 and 15 min of discussions. The sessions address a wide range of medical topics (e.g., general medicine, malaria, HIV–AIDS, diabetes) in French, English, Spanish, and/or Portuguese. The French program is the most complete one with on average 2 h of teaching per week.

#### Post-graduate medical education for residents

The post-graduate medical education (PME) targets residents in universities and teaching hospitals in French-speaking Africa. Video-lectures (45 and 15 min of discussions) are taught by local academic experts and address topics in the field of surgery, ophthalmology, internal medicine, gynecology, pediatrics, etc. The central goal of PME is to produce and share material of high quality for training specialists in Africa.

#### CME and PME: dudal and participation

Video-lectures are produced with Dudal, a distant education software developed for the RAFT to operate with low-bandwidth connections (25 kb/s). It allows participation in educational sessions either as listener or lecturer (12).

Seven hundred thirty-two video-lectures have been broadcast through the RAFT during 2007–2013. Seventy-five, 15, and 10% of them are in French, English, and Spanish, respectively. Today, more than 80% of the French and English video-lectures are produced by African experts. Seventy-five and 25% of them are for CME and PME, respectively. In average, 15 and 3 different sites are connected live during CME and PME video-lectures, respectively, gathering hundreds of health professional in one single video-lecture (in the case of CME).

Although live sessions enable a direct “student–teacher” interaction online, an increasing number of participants choose to watch the pre-recorded video-lectures (possibly because they can be watched at any time of convenience). Lectures are completely free and can be downloaded in several formats.

#### Virtual patients for training on clinical decision-making

Virtual patients are interactive computer simulations of clinical scenarios for medical training (13). Virtual Internet patient simulator (VIPS) is a stand-alone web application initially developed for improving skills of general practitioners in Switzerland, but that was then also used in African countries of the RAFT (14). Studies including African participants, showed that VIPS educational model [paradigm of “blank sheet” (15)] is more suited for learners with some previous experience in the collection of relevant information for decision making (16) and has the potential to improve clinical skills (17).

#### Collaboration with other projects

Africa Build (AB) is a coordination action aiming to support and develop advanced centers of excellence in healthcare, education, and research in the African countries, through Information Technologies (ITs) (18, 19). Dudal was implemented in the AB learning platform (AB Portal) enabling the production and following of courses globally (AB Portal has 14 e-learning courses, 41 discussion groups, and more than 500 users).

Integrated management of childhood illness computerized training tool (ICATT) is an innovative software to support the implementation of the IMCI (WHO/UNICEF strategy) (20). It was developed by the Novartis Foundation in collaboration with the WHO and the Swiss Institute of Tropical Medicine and Public Health. ICATT targets health workers involved in the management of under-5 years children diseases. A multicentric study is currently being developed in Burkina Faso, Cameroon, and Mali to evaluate the impact of ICATT (2 months of self-training with 2 days of clinical practice at the end of fourth week and 2 at the end of eighth week) on acquired skills by health workers and costs compared to traditional training (11 days workshop with 4 days of clinical practice). To support and stimulate learners in ICATT group, e-mail, or short-message service (SMS) reminders are sent at least once a week and the RAFT is used to deliver weekly video-lectures and to foster interactions between them and teachers.

### Clinical and public health activities

#### Tele-expertise activities

Tele-expertise is the second main activity of the RAFT and Bogou is the tool used for it (21). Physicians located in remote and infrastructure limited settings can discuss distantly with specialists from developed areas ensuring a better management of complicated cases (e.g., avoiding unnecessary evacuations). Bogou is a web-based application with secured access through a personal login and password. Medical discussions around cases take place within so called circles (community of recognized professionals). All data in Bogou are encrypted using a public and private key system. Bogou can also operate with digital imaging and communications in medicine (DICOM) images for tele-radiology and tele-ultrasonography.

Overall, there are more than 200 members from 18 countries in Africa, South America, and Asia. There are 22 circles that address cases in general medicine, surgery, infectious diseases, radiology, hematology, endocrinology, cardiology, and gynecology. Since 2012, a total of 471 cases have been posted through Bogou. Promising tele-expertise activities are planned for 2014 in surgery in Ghana and pediatrics in Burkina Faso, Senegal, Ghana, Kenya, and Tanzania, in both cases in the context of different collaborations with two international pharmaceutical companies. In many RAFT countries, these tele-expertise activities are coupled with ultrasound or electrocardiography, etc. For instance, Mali, Cameroon, Ivory Coast, and Madagascar have already implemented tele-ultrasonography and/or tele-cardiology. The large-scale deployment of these activities is planned in Mauritania and Angola with the support of the RAFT.

#### Clinical information systems in African hospitals

There is an increasing demand within the RAFT for the implementation of clinical information systems to improve the collection and management of clinical information in hospitals. Accordingly, two projects based on Open Source systems (22) are in progress at the Mother and Child Hospital in Mali [Cinzan project (22)] and at the Yaoundé Central Hospital in Cameroon [MatLook project (23)].

#### mHealth to support healthcare in Africa

There is an increasing interest in mHealth in public health activities worldwide. There are two mHealth projects in the RAFT: (i) the use of SMS for fast data collection at health district level in Mali and (ii) the impact evaluation of SMS reminders in tuberculosis (TB) cure rate (Cameroon) (24). The objective of this study is to evaluate the effect of SMS reminders on the cure rate of patients with sputum positive pulmonary tuberculosis (TPM+), measured using 6-month bacilloscopy.

#### RAFT Altiplano

The Altiplano project (25, 26) aims at replicating the success of the RAFT in Sub-Saharan Africa in isolated regions in the Bolivian Altiplano. This project builds on previous experiences in Africa and the links between the HUG, UNIGE, and Bolivia in medical informatics. The focal point of this project studied at the University of Geneva where his received a Ph.D. thesis in computer science. The RAFT Bolivian network includes 16 active sites and further extensions will take place in 2014. The tools of the RAFT were translated into Spanish and were setup by the Bolivian technical team.

## Lessons Learnt

Despite the immaturity of activities in some countries (27), the experiences accumulated throughout these years provide both general and specific lessons for each national or local context (28) to promote sustainable implementation of eHealth and telemedicine.

### Social and organization

The development of the RAFT in each country relies primarily on a network of motivated individuals with an interest in eHealth and telemedicine. The RAFT has become an effective multilateral support network in the area of eHealth and telemedicine favoring South–South collaborations for continuous education and management of patients, and the implementation of eHealth activities and telemedicine. Annual coordination workshops with participants from the entire RAFT network are organized in Africa to support the spirit of sharing, strengthen inter-personal links, evaluate the development of the network, and make decisions about future activities (29). Both the social engineering and decentralized management model of the RAFT optimize the management activities (29).

### Human resources

The RAFT builds capacity among health professionals in Africa and supports their activities with different tools (7, 11). In addition, mobility within the RAFT network including the participation in full training programs in eHealth and medical informatics at the UNIGE, has been crucial not only for the participants themselves but also for their colleagues in their home countries. Trained participants act as knowledge multipliers once back in their countries by building competent local teams. This is the case of Mali and Centre d’Expertise et de Recherche en Telemedecine et E-Sante (CERTES). Staffed with a dozen healthcare and IT professionals, the CERTES trains healthcare professionals to use health IT tools, provide operational support for telemedicine activities and health information systems deployment, and run research projects financed by competitive funds (30). This same strategy is also being adopted in Cameroun. The goal is that these centers operate at the subregional level supporting neighbor countries in eHealth and telemedicine activities.

### Software developments and data security

The RAFT tools (Dudal and Bogou) have been developed with Java to run on several operating systems and work reliably with unstable and low-bandwidth connections (e.g., Java web start technology, compression of documents and images, use of text messages for asking questions, data storage under stable and secured environments).

Video-lectures produced with Dudal can be converted to HTML5 making possible to follow them regardless of the web browser. They can also be watched offline after downloading.

Patients data in Bogou are completely secured. Users must go through a validation of their registration demand and then access the system with a username and password. Users must be accepted within a specific circle to be active on the system. Medical data are encrypted via the system of asymmetric encryption public key – private key.

### Policy and legislation

One of the major challenges of the RAFT is to align eHealth and telemedicine activities with local needs and national health strategies. Besides the implication of local actors in the conception and implementation of the activities, the RAFT actively establishes new partnerships with national (e.g., Ministry of Health) and international institutions (e.g., WHO) and/or Non-Governmental Organizations (e.g., UNFM, Agence Universitaire de la Francophonie). This is an anchoring approach both institutional (top-down) and end-user appropriation (bottom-up). The RAFT aims at guiding and supporting countries to progressively implement national strategies, policies, and regulations for eHealth and telemedicine.

Some countries are now developing national eHealth strategies and RAFT local teams are usually involved in this process. In Burkina Faso, the RAFT medical coordinator is now in charge of eHealth at the Ministry of Health. In Ivory Coast, the RAFT focal point has been mandated by the government to lead the development of an eHealth strategy. In Congo-Brazzaville, Mauritania, and Niger, the RAFT model is used as a pilot for the implementation of the national program.

The RAFT also favors the emergence of scientific associations and societies (31, 32) in the context of academia or others sectors that can contribute to the development of eHealth and telemedicine activities and to their transcendence into the national political field. These scientific societies generally led national, regional, or international congresses and workshops gathering the key national scientific and political actors. Mali, Cameroon, Ivory Coast, Tchad, Madagascar among others offer good examples of such societies.

### Economy and financing

#### Financing of the implementation of the RAFT sites and sustainability

After more than a decade of activities, the financial sustainability of the RAFT still remains a major issue (33). Long-term funding is necessary to ensure the involvement of care professionals both as users and providers of quality contents. To motivate a sustainable financial development, the RAFT agrees to provide 2-years worth of connection fees, assuming that the deployment contact will require the receiving hospital to guarantee the financing of these connection costs after the two first years of operation (29). These 2 years are usually sufficient to document the benefits of the activities, and convince decision makers to cover the operational costs. The final goal is to incorporate these activities in governmental plans.

#### Tele-expertise business model

To achieve financial independence of the hospitals and ensure sustainability, a business model is deployed for the tele-expertise service in some of the network nodes, which creates a win–win situation for local physicians as well as the patient. The patient pays for the service locally, thus, avoiding to pay additional transportation costs if it should be done to the capital. Part of this payment is used to finance devices used in the activities or the Internet connection, while the rest is used to pay the treating physician as well as the physician in the reference hospital (29).

Despite the development of these type of business models and their implementation in certain pilot sites, their scaling up is still limited within the RAFT. Numerous institutions are unable to sustainably ensure independent funding for their sites. Business models may be particularly difficult to implement in contexts with low activities. These could be related with the novelty of these types of activities and the immaturity of the telemedicine market in developing countries (34). In these settings, there is still a digital divide and telemedicine targets mainly individuals and not organizations or institutions as in developed countries, which does not allow the economy of scale and higher profits (34). A possible immediate solution could be the creation of national clusters that could drain a critical mass of patients to justify and support the use of resources (equipment, Internet connections, experts’ time, etc.).

However, for effective development of eHealth and telemedicine, it should be put in place a more comprehensive business plan that integrates the context, the needs of stakeholders involved, the technologies-based interventions with high-added value for the improvement of practice and finally, the funding model (35).

## Perspectives

eHealth and telemedicine can potentially improve health systems in LMICs (4, 36–39) and therefore the RAFT has gained interest over the years. The RAFT network counts today with a solid experience in LMICs to keep building on to improve access to quality care.

### Lifelong learning

The RAFT offers health professionals a range of tools and resources (e.g., e-learning material, tele-expertise platform, simulation tools) to ensure their learning through all stages of their career and life. This potentially enhances professional motivation, cohesion, and sustainability of the RAFT network.

### Recognition and valorization

Recognition and valorization of teaching and learning is of major interest in LMICs. Accordingly, Conseil Africain et Malgache pour l’Enseignement Supérieur (CAMES) formally recognizes the production of RAFT courses as an academic merit for career promotion. A new RAFT platform has been recently developed to make the learning experience more complete including learning material to complement video-lectures (e.g., articles, videos) and MCQs. Certificates of participation to the courses can be provided.

However, the main challenge remains to integrate these contents in formal programs of continued education or of post-graduate studies with a full recognition in form of ECTS credits.

### Impact evaluation

Impact evaluation of the RAFT activities remains an “unfinished business” (40–43), and therefore, it must be the priority for the next years. We aim at measuring the impact of the RAFT on clinical (patient), public health (population, health system), and socio-economic (patient, health system) domains, in terms of processes and outcomes [e.g., Mali (44), Cameroon]. This goal is also the major demand by decision makers and donors (45–49). To achieve this one, it is required to develop good methodologies for optimal impact assessment of eHealth and telemedicine activities before.

### Scaling up and transferability out of Africa

Although the RAFT is active in a considerable number of countries of the world, the number of sites per country is low and they are mainly concentrated in reference hospitals in urban areas. A larger number of sites and a wider distribution in the periphery are needed to improve health systems.

Due to infrastructure and economic reasons, the best level of deployment is the district hospital. District hospitals generally cover an important population (50,000–200,000 inhabitants), include a critical mass of health professionals (2 or more physicians) and have medico-technical infrastructure such as laboratories and operating room and associated activities that cover primary cares. 3G Internet connections can be easily obtained for reasonable costs.

The RAFT is built on basic and general principles and ways of operation that respond to important and common needs around the globe, making it potentially transferable. The success of the RAFT in the Bolivian Altiplano and the promising progresses made in Nepal during the last months evidence that RAFT is not only African but also potentially global. However, possible local needs and socio-cultural and political differences even within the same geographical sub-region must be carefully considered and adapted to them.

## Conflict of Interest Statement

The authors declare that the research was conducted in the absence of any commercial or financial relationships that could be construed as a potential conflict of interest.

## Footnotes

^1^http://raft.g2hp.net/sites/

## References

[1] Organisation Mondiale de la Santé. La crise des personnels de la Santé (2014). Available from: http://www.who.int/hrh/strategy/information/fr/

[2] Sikwese A Mwape L Mwanza J Kapungwe A Kakuma R Imasiku M Human resource challenges facing Zambia’s mental health care system and possible solutions: results from a combined quantitative and qualitative study. Int Rev Psychiatry (2010) 22:550–7 10.3109/09540261.2010.536148 21226643

[3] Dzenowagis J Bridging the digital divide. In: Wootton R Patil NG Scott RE Ho K, editors. Telehealth in the Developing World. London: Royal Society of Medicine Press (2009). p. 9–25

[4] Blaya JA Fraser HS Holt B e-Health technologies show promise in developing countries. Health Aff (Millwood) (2010) 29:244–51 10.1377/hlthaff.2009.0894 20348068

[5] Rao B Lombardi A Telemedicine: current status in developed and developing countries. J Drugs Dermatol (2009) 8:371–5 19363855

[6] Hasham S Hasham S Implementation lessons on the use of innovation in information technology in the developing world. World Hosp Health Serv (2013) 49:10–3 24377141

[7] Bagayoko CO Anne A Fieschi M Geissbuhler A Can ICTs contribute to the efficiency and provide equitable access to the health care system in Sub-Saharan Africa? The Mali experience. Yearb Med Inform (2011) 6(1):33–8 21938322

[8] RAFT. (2014). Available form: http://raft.hcuge.ch/

[9] Bagayoko CO Muller H Geissbuhler A Assessment of Internet-based tele-medicine in Africa (the RAFT project). Comput Med Imaging Graph (2006) 30:407–16 10.1016/j.compmedimag.2006.09.014 17049808

[10] Geissbuhler A Bagayoko CO Ly O The RAFT network: 5 years of distance continuing medical education and tele-consultations over the Internet in French-speaking Africa. Int J Med Inform (2007) 76:351–6 10.1016/j.ijmedinf.2007.01.012 17331799

[11] Bagayoko CO Perrin C Gagnon MP Geissbuhler A Continuing distance education: a capacity-building tool for the de-isolation of care professionals and researchers. J Gen Intern Med (2013) 28(Suppl 3):666–70 10.1007/s11606-013-2522-1 23780652 PMC3744283

[12] Dudal. (2014). Available from: http://raft.unige.ch/dudal/

[13] MedBiquitous Virtual Patient. Specifications and Description Document (2014). Available from: http://www.medbiq.org

[14] Bediang G Bagayoko CO Raetzo MA Geissbuhler A Relevance and usability of a computerized patient simulator for continuous medical education of isolated care professionals in Sub-Saharan Africa. Stud Health Technol Inform (2011) 169:666–70 21893831

[15] Nendaz MR Raetzo MA Junod AF Vu NV Teaching diagnostic skills: clinical vignettes or chief complaints? Adv Health Sci Educ Theory Pract (2000) 5:3–10 10.1023/A:1009887330078 12386472

[16] Bediang G Raetzo MA Geissbuhler A Virtual patient simulation: a comparison of two approaches for capacity building in Sub-Saharan Africa. Stud Health Technol Inform (2012) 180:978–82 22874339

[17] Bediang G Franck C Raetzo MA Doell J Ba M Kamga Y Developing clinical skills using a virtual patient simulator in a resource-limited setting. Stud Health Technol Inform (2013) 192:102–6 23920524

[18] Africa Build Project. (2014). Available from: http://www.africabuild.eu/

[19] Jimenez-Castellanos A Ramirez-Robles M Shousha A Bagayoko CO Perrin C Zolfo M Enhancing research capacity of African institutions through social networking. Stud Health Technol Inform (2013) 192:1099 23920873

[20] ICATT (Integrated Management of Childhood Illness Computerized Adaptation and Training Tool). (2014). Available from: http://www.icatt-training.org/

[21] Bogou. (2014). Available from: http://raft.unige.ch/bogou/

[22] Bagayoko CO Dufour JC Chaacho S Bouhaddou O Fieschi M Open source challenges for hospital information system (HIS) in developing countries: a pilot project in Mali. BMC Med Inform Decis Mak (2010) 10:22 10.1186/1472-6947-10-22 20398366 PMC2868794

[23] MatLook. (2014). Available from: http://matlook.net

[24] Bediang G Stoll B Elia N Abena JL Nolna D Chastonay P SMS reminders to improve the tuberculosis cure rate in developing countries (TB-SMS Cameroon): a protocol of a randomised control study. Trials (2014) 15:35 10.1186/1745-6215-15-35 24460827 PMC3902069

[25] RAFT Altiplano. (2014). Available from: http://raft-altiplano.blogspot.ch/

[26] Sierro F Adam Y Musso P May A Projet RAFT-Altiplano: la télémédecine en Bolivie. Rapport de stage d’immersion en communauté 2012 (2014). Available from: http://www.medecine.unige.ch/enseignement/apprentissage/module4/immersion/archives/2011_2012/rapports/Projet_RAFT_ALTIPLANO_%20la%20t%C3%A9l%C3%A9m%C3%A9decine_en_Bolivie.pdf

[27] Shiferaw F Zolfo M The role of information communication technology (ICT) towards universal health coverage: the first steps of a telemedicine project in Ethiopia. Glob Health Action (2012) 5:1–8 10.3402/gha.v5i0.15638 22479235 PMC3318899

[28] Lam CL Knowledge can flow from developing to developed countries. BMJ (2000) 321:830 10.1136/bmj.321.7264.830 11009533 PMC1118628

[29] Franck C Bediang G Wac K Bagayoko CO Geissbuhler A Health workforce capacity building in Africa with support of ICT: lessons learnt and key challenges. In: Proceedings of the IEEE Healthcare Technology Conference: Translational Engineering in Health & Medicine Houston, TX (2012).

[30] Otero PD Perrin C Geissbuhler A Cheung NT Theera-Ampornpunt N Lun KC Informatics education in low-resources settings. In: Berner ES, editor. Informatics Education in Healthcare. London: Springer (2014). p. 197–222

[31] Société Malienne d’Informatique Biomédicale et de santé. (2014). Available from: http://www.somibs.net/

[32] Société Camerounaise d’Informatique Médicale et de Télémédecine. (2014). Available from: http://www.socim.cm/

[33] Bediang G Bagayoko CO Geissbuhler A Medical decision support systems in Africa. Yearb Med Inform (2010):47–54 20938570

[34] Chen S Cheng A Mehta K A review of telemedicine business models. Telemed J E Health (2013) 19:287–97 10.1089/tmj.2012.0172 23540278

[35] van LM van Gemert-Pijnen JE Nijland N Ossebaard HC Hendrix RM Seydel ER Why business modeling is crucial in the development of eHealth technologies. J Med Internet Res (2011) 13:e124 10.2196/jmir.1674 22204896 PMC3278110

[36] Heeks R Do information and communication technologies (ICTs) contribute to development? J Int Dev (2010) 22:625–40 10.1002/jid.1716

[37] Department for International Developement (London). Building the Evidence to Reduce Poverty (2014). Available from: http://www.gov.uk/government/uploads/system/uploads/attachment_data/file/67729/evaluation-policy.pdf

[38] Wamala DS Augustine K A meta-analysis of telemedicine success in Africa. J Pathol Inform (2013) 4:6 10.4103/2153-3539.112686 23858382 PMC3709418

[39] Mbarika VWA Okoli C Telemedicine in Sub-Saharan Africa: A Proposed Delphi Study. In: Proceedings of the 36th Hawaii International Conference on System Sciences (HICSS’03) Baton Rouge, LA: IEEE (2003). 10.1109/HICSS.2003.1174373

[40] Nassar BS Vaughan-Sarrazin MS Jiang L Reisinger HS Bonello R Cram P Impact of an intensive care unit telemedicine program on patient outcomes in an integrated health care system. JAMA Intern Med (2014) 174:1160–7 10.1001/jamainternmed.2014.1503 24819673 PMC13003558

[41] Mechael P Nemser B Cosmaciuc R Cole-Lewis H Ohemeng-Dapaah S Dusabe S Capitalizing on the characteristics of mHealth to evaluate its impact. J Health Commun (2012) 17(Suppl 1):62–6 10.1080/10810730.2012.679847 22548600

[42] Chang LW Kagaayi J Arem H Nakigozi G Ssempijja V Serwadda D Impact of a mHealth intervention for peer health workers on AIDS care in rural Uganda: a mixed methods evaluation of a cluster-randomized trial. AIDS Behav (2011) 15:1776–84 10.1007/s10461-011-9995-x 21739286 PMC3265752

[43] Long MC Angtuaco T Lowery C Ultrasound in telemedicine: its impact in high-risk obstetric health care delivery. Ultrasound Q (2014) 30:167–72 10.1097/RUQ.0000000000000073 25148484

[44] Bagayoko CO Traore D Thevoz L Diabate S Pecoul D Niang M Medical and economic benefits of telehealth in low- and middle-income countries: results of a study in four district hospitals in Mali. BMC Health Serv Res (2014) 14(Suppl 1):S9 10.1186/1472-6963-14-S1-S9 25080312 PMC4108933

[45] Piette JD Lun KC Moura LA Jr Fraser HS Mechael PN Powell J Impacts of e-health on the outcomes of care in low- and middle-income countries: where do we go from here? Bull World Health Organ (2012) 90:365–72 10.2471/BLT.11.099069 22589570 PMC3341688

[46] Rigby M Impact of telemedicine must be defined in developing countries. BMJ (2002) 324:47–8 10.1136/bmj.324.7328.47a 11797639 PMC1121950

[47] Roine R Ohinmaa A Hailey D Assessing telemedicine: a systematic review of the literature. CMAJ (2001) 165(6):765–71 11584564 PMC81454

[48] Ekeland AG Bowes A Flottorp S Effectiveness of telemedicine: a systematic review of reviews. Int J Med Inform (2010) 79:736–71 10.1016/j.ijmedinf.2010.08.006 20884286

[49] Scott R.E Saeed A Global eHealth-Measuring Outcomes: Why, What and How. A Report Commissioned by the WHO Global Observatory for eHealth. Bellagio (2008). Available from: http://www.ehealth-connection.org/files/conf-materials/Global%20eHealth%20-%20Measuring%20Outcomes_0.pdf

